# Ocular motor cranial neuropathy and risk of thyroid cancer: A Korean population-based study

**DOI:** 10.1371/journal.pone.0319872

**Published:** 2025-03-12

**Authors:** Jaeryung Kim, Kyung-Ah Park, Kyungdo Han, Jin-Hyung Jung, Sei Yeul Oh, Soolienah Rhiu

**Affiliations:** 1 Department of Ophthalmology, Samsung Medical Center, Sungkyunkwan University School of Medicine, Seoul, Republic of Korea; 2 Department of Statistics and Actuarial Science, Soongsil University, Seoul, Republic of Korea; 3 Samsung Biomedical Research Institute, Sungkyunkwan University School of Medicine, Suwon, Republic of Korea; 4 Department of Ophthalmology, Hallym University School of Medicine, Dongtan Sacred Heart Hospital, Hwaseong, Republic of Korea; West China Hospital of Sichuan University, CHINA

## Abstract

This study investigates whether ocular motor cranial neuropathy (OMCN) can predict the onset of thyroid cancer given its association with common cardiovascular risk factors including obesity, diabetes mellitus (DM), hypertension, and dyslipidemia. We conducted a retrospective, nationwide, population-based cohort study utilizing data from the Korean National Health Insurance Service. Individuals comprised those aged ≥ 20 years diagnosed with OMCN between 2010 and 2017. Exclusions were based on pre-existing conditions, inability to match controls, or incomplete data. The study involved 118,686 participants, with 19,781 in the OMCN group and 98,905 controls, matched for age and sex. Patients with OMCN showed a significantly higher risk of developing thyroid cancer (hazard ratio [HR] =  1.392 [95% confidence interval, 1.075–1.802]) compared to controls after adjusting for potential confounding factors. This association was more pronounced in participants with DM and women (HR =  2.288 in DM vs. HR =  1.209 in non-DM, p =  0.0450; HR =  1.677 in females vs. HR =  0.824 in males, p =  0.0246). The findings suggest OMCN as a potential early predictor of thyroid cancer risk, particularly in diabetic and female patients. Further research is needed to explore the underlying mechanisms linking cardiovascular risk factors to the relationship between OMCN and thyroid cancer. Proactive management of these risk factors in OMCN patients may contribute to thyroid cancer prevention.

## Introduction

Ocular motor cranial neuropathy (OMCN), a condition that impairs the functioning of one or more of the cranial nerves governing ocular movement, is frequently encountered in ophthalmology practice [1–5], with incidence rates of 4.0 per 100,000 person-years for third [2], 5.7 per 100,000 person-years for fourth [1], and 11.3 cases per 100,000 person-years for sixth cranial nerve palsy (CNP) [6]. The manifestation of OMCN is typically characterized by abrupt ocular deviation and diplopia, together with various related symptoms depending on the etiology and location of the lesion [1,7–9], potentially facilitating its prompt diagnosis. The main pathogenesis of OMCN is regarded as atherosclerotic changes in small blood vessels, while the risk of its development has been reported to be associated with several risk factors, including mechanical causes like head trauma, infections, and space-occupying lesions, as well as cardiovascular risk factors such as obesity, diabetes mellitus (DM), hypertension, and dyslipidemia [2–4,10,11].

The incidence of thyroid cancer, a malignancy originating from the thyroid parenchymal cells, has consistently increased globally [12], making it the 11th most prevalent cancer worldwide according to GLOBOCAN 2018 estimates of cancer incidence and mortality from the International Agency for Research on Cancer [13]. Although the precise causes remain elusive, several studies have explored potential risk factors, including exposure to radiation, genetic alterations, sex (with a higher prevalence in women), and iodine-deficient diet [12,14,15]. Of particular relevance to our study, recent evidence has demonstrated strong associations between thyroid cancer risk and cardiovascular risk factors like obesity [14,16,17], DM [18], and dyslipidemia [19]. Conversely, thyroid cancer patients have been reported to have a higher risk of cardiovascular diseases [20–23], suggesting a complex relationship between cardiovascular risk factors and thyroid cancer.

Given this interplay between cardiovascular risk factors and thyroid cancer, along with recent evidence suggesting OMCN as a predictor of diseases associated with cardiovascular risk factors [24], we hypothesized that well-documented cardiovascular risk of OMCN patients might have implications for thyroid cancer development. Thus, this research aimed to investigate the association between OMCN and thyroid cancer risk after adjusting for potential confounders, with the goal of improving early detection strategies in this patient population.

## Methods

### Data sources and study population

In this study, we used data from the Korean National Health Insurance Service (KNHIS), which covers approximately 97% of the Korean population [25]. Anonymized health examination data, including laboratory results, anthropometric measurements, self-reported questionnaire responses, health-related behaviors, and disease diagnoses using Korean Standard Classification of Diseases seventh revision (KCD-7) codes in alignment with the International Classification of Diseases 10^th^ revision (ICD-10) system, were sourced from data of the biennial Korean National Health Screening Program (NHSP) for individuals aged 20 years and older courtesy of the KNHIS. Throughout the study, the authors had no access to any information that could identify individual participants. These data are publicly available for all researchers receiving approval by their institutional review board (IRB).

**Fig 1:**
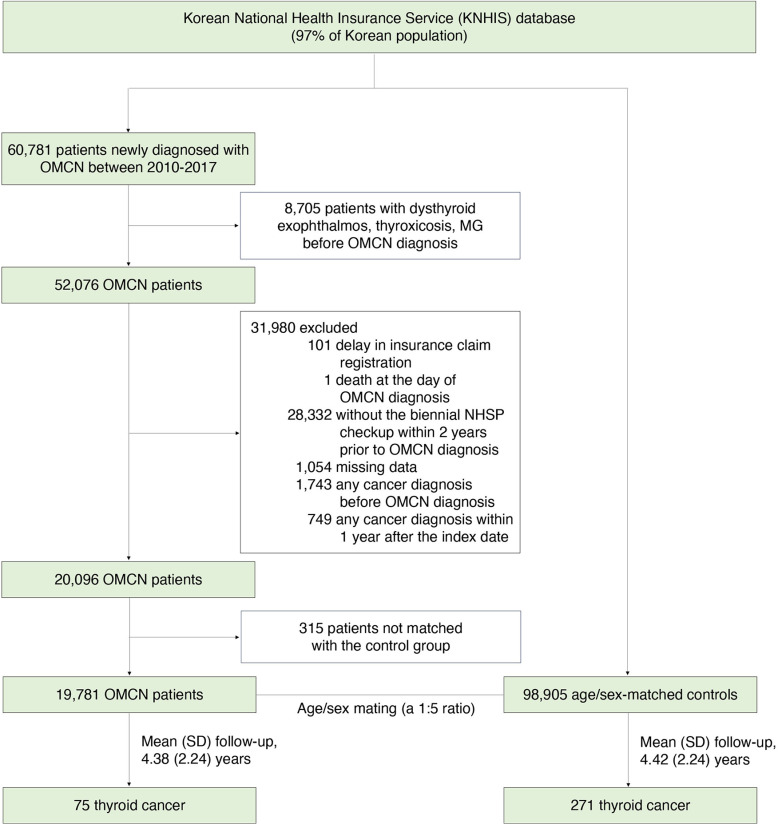
Flowchart illustrating the selection of study participants. KNHIS, Korean National Health Insurance Service; MG, myasthenia gravis; NHSP, National Health Screening Program; OMCN, ocular motor cranial neuropathy; SD, standard deviation.

During 2010–2017, a total of 60,781 cases of OMCN was identified based on the ICD-10 codes of OMCN H49.0 (third CNP), H49.1 (fourth CNP), or H49.2 (sixth CNP). Detailed health examination data, including information on smoking and drinking status, height, weight, body mass index (BMI), waist circumference (WC), serum glucose and cholesterol levels, and systolic and diastolic blood pressures (BPs) were obtained. Several exclusion criteria were applied. Participants who had been diagnosed with dysthyroid exophthalmos (H06.2), thyrotoxicosis (E05), or myasthenia gravis (G70.0) prior to their diagnosis with OMCN were excluded (n =  8,705). Additionally, individuals whose insurance claim registrations were delayed (n =  101), those who died on the day of OMCN diagnosis (n =  1), and those who did not undergo the NHSP survey within two years before the diagnosis of OMCN (n =  28,332) were excluded. Furthermore, participants with missing health examination data were also excluded (n =  1,054). To minimize the influence of other pre-existing diseases, individuals with a history of cancer before cohort entry (n =  1,743) and those who were newly diagnosed with any type of cancer within one year after OMCN diagnosis (n =  749) were excluded. Five age- and sex-matched controls were selected for every OMCN case. Participants who were not matched by age and sex with the control group were excluded (n =  315). Ultimately, this study included 19,781 OMCN cases and 98,905 controls (Fig 1).

### Ethical statement

The study adhered to the tenets of the Declaration of Helsinki and Strengthening the Reporting of Observational Studies in Epidemiology reporting guidelines and was approved by the IRB of Samsung Medical Center (IRB no. 2023-05-099), which exempted the need for individual patient informed consent due to the study’s retrospective design and adherence to confidentiality protocols while using anonymized public data.

### Measurements, definitions, and study endpoints

Variables of sex, age, smoking status, amount of alcohol consumption, regular exercise, income status, and obesity were considered as confounders. Clinical variables were defined using standardized criteria established in our previous KNHIS-based studies [11,19,24]. In brief, individuals indicating daily tobacco use or any consumption of pure alcohol were labeled as current smokers or drinkers, respectively. Those confirming their status as either non-smokers or former smokers were grouped as non-smokers, and those who stated current abstention from alcohol were designated as non-drinkers. We classified participants as regular exercisers if they reported either vigorous physical activity lasting at least 20 minutes three or more times weekly or moderate physical activity lasting 30 minutes or longer five or more times weekly. We stratified income levels into four quartiles, with the lowest quartile of the entire population corresponding to the low-income level. BMI was determined by dividing an individual’s weight (in kg) by the square of their height (in meters). To determine obesity among the Korean participants in our study, we used a threshold of 25 kg/m^2^, as recommended in the Overweight and Obesity Clinical Practice Guidelines set forth by the Korean Society for the Study of Obesity. The analysis also accounted for several key medical conditions. We identified DM based on either laboratory values (fasting glucose ≥ 126 mg/dL) or medical records showing prescriptions for diabetes medications (oral agents or insulin) with corresponding diagnostic codes (E10-14). We classified participants as having hypertension if they had elevated blood pressure readings (systolic ≥ 140 mmHg or diastolic ≥ 90 mmHg) or if their records showed antihypertensive medication prescriptions along with relevant diagnostic codes (I10-13, I15). Participants met the criteria for dyslipidemia if they had elevated total cholesterol (≥240 mg/dL) or were receiving lipid-lowering medications with associated diagnostic codes (E78). Chronic kidney disease (CKD) classification was based on estimated glomerular filtration rates below 60 mL/min/1.73 m².

The primary outcome was newly diagnosed thyroid cancer cases, defined by a new ICD-10 claim for thyroid cancer (C73) in conjunction with the KNHIS’s cancer-specific insurance claim code V193. To minimize concerns of reverse causality, we implemented a one-year lag period from the index date, excluding all cancer diagnoses including thyroid cancer during this period. Follow-up continued until thyroid cancer diagnosis, death, or the study’s end date (December 31, 2019), whichever occurred first.

### Statistical analysis

Baseline characteristics are presented as percentage (%) for categorical data or mean with standard deviation for continuous data. We employed Student’s *t* test to assess continuous data and the chi-square test for categorical data differences. We applied a Cox proportional hazards regression method with adjustments for multiple variables to determine hazard ratios (HRs) along with their 95% confidence intervals (CIs) to study the potential relationship between OMCN and the risk of thyroid cancer. Model 1 had no adjustments, whereas model 2 was refined for age and sex factors. Model 3 incorporated the variables present in model 2 together with smoking habits, alcohol intake, regular physical activity, income quartile, and BMI. Finally, model 4 expanded upon model 3 by integrating comorbidity variables of DM, hypertension, dyslipidemia, and CKD. Additionally, we calculated HRs with 95% CIs for OMCN presence, accounting for the same confounding factors as in model 4. For those with OMCN compared to those not having it, we determined HRs and 95% CIs, classifying them based on sex, age group (20–39, 40–64, or ≥ 65 years), smoking status, alcohol usage, regular exercise, income quartile, obesity, DM, hypertension, and dyslipidemia. All data analyses were performed using SAS version 9.4 (SAS Institute Inc., Cary, NC, USA), and p <  0.05 was considered statistically significant.

## Results

### Baseline characteristics of the study population

Baseline characteristics of the study population are shown in Table 1. Compared to controls, individuals with OMCN exhibited less frequent alcohol consumption; a smaller proportion belonging to the lowest income quartile; and higher prevalence rates of obesity, DM, hypertension, and dyslipidemia. They also had higher BMI, WC, glucose level, and systolic and diastolic BP values compared to the control group.

**Table 1:** Baseline characteristics of subjects without and with OMCN.

Variable	Total (N = 118,686)	Non-OMCN (N = 98,905)	OMCN (N = 19,781)	p value
Age (years)	59.82 ± 13.05	59.82 ± 13.05	59.82 ± 13.05	1
20-39	9,282 (7.82%)	7,735 (7.82%)	1,547 (7.82%)	1
40-64	62,010 (52.25%)	51,675 (52.25%)	10,335 (52.25%)	
65-	47,394 (39.93%)	39,495 (39.93%)	7,899 (39.93%)	
Sex (male)	74,778 (63.00%)	62,315 (63.00%)	12,463 (63.00%)	1
Current smoker	26,318 (22.17%)	22,014 (22.26%)	4,304 (21.76%)	0.1227
Current drinker	51,462 (43.36%)	43,451 (43.93%)	8,011 (40.5%)	<0.0001
Regular physical activity	25,379 (21.38%)	21,104 (21.34%)	4,275 (21.61%)	0.3909
Income, low	23,973 (20.20%)	20,126 (20.35%)	3,847 (19.45%)	0.0040
Obesity	44,288 (37.32%)	36,386 (36.79%)	7,902 (39.95%)	<0.0001
DM	21,978 (18.52%)	16,029 (16.21%)	5,949 (30.07%)	<0.0001
Hypertension	53,620 (45.18%)	43,508 (43.99%)	10,112 (51.12%)	<0.0001
Dyslipidemia	39,819 (33.55%)	31,729 (32.08%)	8,090 (40.90%)	<0.0001
CKD	24.13 ± 3.15	7,350 (7.43%)	1,620 (8.19%)	0.0002
BMI (kg/m^2^)	24.13 ± 3.15	24.09 ± 3.13	24.35 ± 3.24	<0.0001
WC (cm)	82.83 ± 8.83	82.69 ± 8.78	83.56 ± 9.03	<0.0001
Fasting glucose (mg/dL)	104.34 ± 29.94	102.75 ± 26.68	112.29 ± 41.74	<0.0001
Systolic BP (mmHg)	125.77 ± 15.33	125.64 ± 15.26	126.43 ± 15.64	<0.0001
Diastolic BP (mmHg)	77.29 ± 9.97	77.23 ± 9.91	77.6 ± 10.24	<0.0001
Total cholesterol (mg/dL)	195.22 ± 38.36	195.52 ± 38.07	193.71 ± 39.77	<0.0001

Categorical data are presented in percentage (%) form, while continuous data are given as mean ±  standard deviation. For continuous variables, p-values were derived using Student’s t test; for categorical variables, the chi-square test was employed. OMCN: ocular motor cranial neuropathy; N: number; DM: diabetes mellitus; CKD: chronic kidney disease; BMI: body mass index; WC: waist circumference; BP: blood pressure.

### Risk of thyroid cancer according to the presence of OMCN

**Fig 2:**
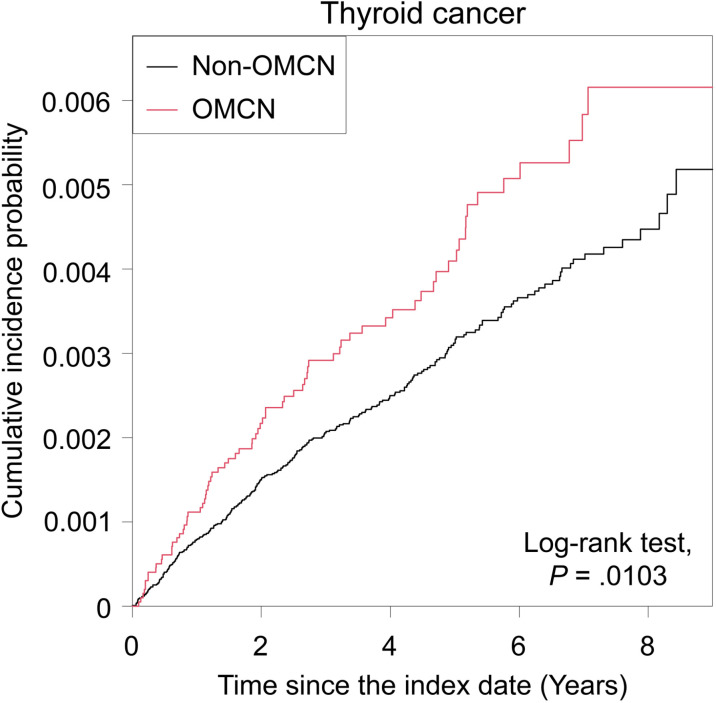
The cumulative incidence of thyroid cancer stratified by ocular motor cranial neuropathy status.

During the follow-up period, a total of 346 cases was diagnosed with thyroid cancer considering a lag period of one year (Table 2). In comparison to the control group, patients with OMCN had a higher risk of thyroid cancer. The incidence rate (IR) of thyroid cancer was 0.620 cases per 1,000 person-years in the control group, while it was 0.867 cases per 1,000 person-years in the OMCN group. After adjusting for various factors, the HR in model 4 was 1.392 (95% CI, 1.075–1.802), indicating an increased risk of thyroid cancer among patients with OMCN (Table 2). The Kaplan–Meier curve for incidence probability of thyroid cancer according to OMCN is shown in Fig 2, suggesting that the OMCN group had a higher incidence probability of thyroid cancer (p =  0.0103).

**Table 2:** Analysis of thyroid cancer incidence using multivariable-adjusted Cox regression in relation to OMCN presence.

OMCN	N	Event	Person-years	IR	HR (95% CI)			
					Model 1^a^	Model 2^b^	Model 3^c^	Model 4^d^
No	98,905	271	436,793.3	0.620	1 (reference)	1 (reference)	1 (reference)	1 (reference)
Yes	19,781	75	86,542.29	0.867	1.396 (1.081–1.802)	1.397 (1.081–1.803)	1.387 (1.074–1.791)	1.392 (1.075–1.802)
p-value					0.0106	0.0105	0.0122	0.0122

^a^Without adjusting factors;

^b^Adjusted for age and sex;

^c^Adjusted for age, sex, smoking, alcohol consumption, physical activity, income status, and obesity;

^d^Adjusted for age, sex, smoking, alcohol consumption, physical activity, income status, obesity, diabetes mellitus, hypertension, dyslipidemia, and CKD. OMCN: ocular motor cranial neuropathy; N: number; IR: incidence rate per 1,000 person-years; HR: hazard ratio; CI: confidence interval; CKD: chronic kidney disease.

### Subgroup analysis

Table 3 presents multivariable-adjusted HRs for the emergence of thyroid cancer; segmented by factors of sex, age group, smoking habit, alcohol intake, physical exercise, income status, and comorbidities (i.e., obesity, DM, hypertension, and dyslipidemia). A stronger association between OMCN and thyroid cancer development was observed in the female group (HR =  1.677 [95% CI, 1.250–2.251], *P* for interaction = .0246) compared to the male group. Except for DM (HR =  2.288 [95% CI, 1.325–3.949; *P* for interaction = .0450), no notable synergistic interactions were identified between OMCN and other variables, especially major cardiovascular risk factors like hypertension, dyslipidemia, and obesity, in relation to thyroid cancer HRs.

**Table 3:** Multivariable-adjusted Cox regression analysis demonstrating an association between OMCN and development of thyroid cancer based on other characteristics of subjects.

Subgroup		OMCN	HR (95% CI)	p for interaction
Sex	Male	No	1 (reference)	0.0246
		Yes	0.824 (0.476–1.426)	
	Female	No	1 (reference)	
		Yes	1.677 (1.250–2.251)	
Age (years)	20-39	No	1 (reference)	0.7327
		Yes	1.857 (0.863–3.995)	
	40-64	No	1 (reference)	
		Yes	1.359 (0.978–1.887)	
	65-	No	1 (reference)	
		Yes	1.310 (0.799–2.146)	
Smoking	No	No	1 (reference)	0.0752
		Yes	1.522 (1.162–1.993)	
	Yes	No	1 (reference)	
		Yes	0.632 (0.249–1.604)	
Alcohol consumption	No	No	1 (reference)	0.0684
		Yes	1.629 (1.207–2.198)	
	Yes	No	1 (reference)	
		Yes	0.937 (0.559–1.570)	
Regular physical activity	No	No	1 (reference)	0.8803
		Yes	1.377 (1.027–1.846)	
	Yes	No	1 (reference)	
		Yes	1.443 (0.841–2.475)	
Income status	Other	No	1 (reference)	0.3371
		Yes	1.298 (0.963–1.751)	
	Low quartile 1	No	1 (reference)	
		Yes	1.730 (1.042–2.875)	
Obesity	No	No	1 (reference)	0.2117
		Yes	1.197 (0.837–1.714)	
	Yes	No	1 (reference)	
		Yes	1.661 (1.145–2.411)	
Diabetes mellitus	No	No	1 (reference)	0.0450
		Yes	1.209 (0.894–1.637)	
	Yes	No	1 (reference)	
		Yes	2.288 (1.326–3.949)	
Hypertension	No	No	1 (reference)	0.8402
		Yes	1.425 (1.007–2.017)	
	Yes	No	1 (reference)	
		Yes	1.352 (0.921–1.983)	
Dyslipidemia	No	No	1 (reference)	0.0884
		Yes	1.164 (0.828–1.638)	
	Yes	No	1 (reference)	
		Yes	1.838 (1.229–2.749)	
Chronic kidney disease	No	No	1 (reference)	0.1363
		Yes	1.466 (1.127–1.907)	
	Yes	No	1 (reference)	
		Yes	0.476 (0.111–2.045)	

^a^Adjusted for age, sex, smoking status, alcohol consumption, physical activity, income status, obesity, diabetes mellitus, hypertension, and dyslipidemia. OMCN: ocular motor cranial neuropathy; HR: hazard ratio; CI: confidence interval.

## Discussion

In this study, we demonstrate that OMCN is associated with an elevated risk of thyroid cancer using a nationwide, population-based cohort of Koreans. This association remained significant even after adjusting for potential confounders of age, sex, lifestyle behaviors (smoking and alcohol intake), amount of physical activity, income, and existing health conditions (obesity, DM, hypertension, and dyslipidemia). Of note, the data revealed a more prominent risk of thyroid cancer in association with OMCN among women or those with DM. To the best of our knowledge, this study is the first in-depth exploration of the link between OMCN and thyroid cancer risk in a nationwide, population-based context.

The primary pathogenesis of OMCN is associated with compromised cardiovascular system, although various etiologies, including trauma, neoplasms, aneurysms, and other diverse causes, also contribute [26,27]. The incidence of OMCN has been reported to be higher in patients with metabolic syndrome, in which the main components include insulin resistance, hypertension, hypertriglyceridemia, low levels of high-density lipoprotein cholesterol, and obesity [11]. Of note, OMCN functions as a strong early predictor for the onset of vascular dementia [24], which results from diminished blood flow to the brain secondary to cardiovascular and cerebrovascular diseases. This result suggests that OMCN may play a pivotal role in predicting the development of diseases associated with compromised cardiovascular system, especially given the prompt diagnosis of OMCN due to its distinct and acute presentation. Concurrently, prior studies have demonstrated a significant correlation between compromised cardiovascular system and an increased risk of subsequent cancer development [28,29]. Our findings extend this predictive value of OMCN to thyroid cancer risk, particularly in the context of shared cardiovascular risk factors including obesity, DM, hypertension, and dyslipidemia.

Studies have established robust association between compromised cardiovascular system and thyroid cancer development [14,16–19,30]. Concerning obesity, a meta-analysis encompassing seven cohort studies revealed that obesity heightened the risk of thyroid cancer by 18% [16]. Obese individuals exhibited a notably greater risk of thyroid cancer compared to their normal-weight counterparts [17]. Additionally, a hospital-based case–control study identified a positive association between elevated BMI and an augmented risk of thyroid cancer [14]. In relation to DM, the NIH-AARP Diet and Health Study demonstrated that diabetic women hold a significantly elevated risk of differentiated thyroid cancer [18]. Regarding dyslipidemia, persistently low high-density lipoprotein cholesterol levels across four consecutive health checkups correlated with a heightened thyroid cancer risk [19]. Intriguingly, patients with thyroid cancer from the Surveillance, Epidemiology, and End Results database showed that 29.1% of deaths were attributable to thyroid cancer itself, while 21.7% were due to cardiovascular disease [20]. Some studies propose that thyroid cancer management might correlate with elevated risks of cardiovascular disease [22] and atrial fibrillation [21] compared to a general healthy population. Upon age stratification, thyroid cancer survivors, particularly those < 65 years of age, consistently exhibited a marked coronary heart disease risk even during extended post-treatment periods [30]. A comprehensive meta-analysis of 15 studies with a cumulative 771,220 post-thyroidectomy patients showed a higher risk for both cerebrovascular diseases and atrial fibrillation [23]. Collectively, these cardiovascular risk factors exhibit a robust association with thyroid cancer risk through intricate inter-relationships.

In this study, we found that OMCN was significantly associated with an increased risk of thyroid cancer, even after adjusting for potential confounding factors. A possible explanation for the association between OMCN and thyroid cancer is that the two conditions involve similar underlying mechanisms related to shared cardiovascular risk factors. Although the mechanism of the increased risk of thyroid cancer by cardiovascular diseases needs to be further elucidated, we postulate that there are common biological processes like inflammation and metabolic adaptations. Recently, murine studies have investigated the relationship between cardiovascular diseases and the risk of cancer. Obesity mouse models showed that persistent inflammation in white adipose tissue can promote cancer development due to changes in levels of inflammatory mediators and adipokines and increased insulin resistance [31]. In addition, heart failure models in mice showed increased cancer formation through secreted circulating factors [32,33], while another study on acute myocardial infarction in mice demonstrated cancer progression via reprogramming of the innate immune system [34]. Furthermore, human genetic studies identified a significant atherosclerosis-associated variant in a known cancer locus [35,36]. In the context of cardiovascular diseases and cancer, certain therapies developed for one disease have also shown beneficial effects on the other [37,38]. Meanwhile, we observed a stronger association between OMCN and the risk of thyroid cancer in patients with DM and in female patients. While the reason why female OMCN patients have a higher risk of thyroid cancer compared to male OMCN patients is not known, one hypothesis is that female OMCN patients access healthcare services more frequently. This enhanced utilization may elevate the risk of overdetection of subclinical thyroid cancer, consistent with prior observations [39]. Indeed, while there was no difference in detection rates of early-stage subclinical thyroid cancer between men and women at autopsy, the identification of subclinical thyroid cancer during one’s lifetime was approximately four times more prevalent in women than in men, likely due to overdiagnosis [39]. A significant additive interaction was also observed between OMCN and DM in relation to the risk of thyroid cancer development. Additionally, there was a trend toward an additive interaction between OMCN and other cardiovascular risk factors, such as dyslipidemia and obesity, although these relationships were not statistically significant. These findings underscore the importance of managing cardiovascular risk factors in the prevention and early detection of thyroid cancer.

Several important limitations warrant consideration when interpreting our findings. The retrospective design inherently prevents establishing direct causality between OMCN and thyroid cancer, limiting our conclusions to associative relationships. A significant methodological constraint stems from our reliance on the NHSP health screening program, which captures approximately 70-80% of eligible individuals [40]. This sampling approach may introduce selection bias, as participants likely represent a more health-conscious subset of the population, particularly given the program’s non-significant incentive structure for medical cost reduction. Our use of diagnostic codes for identifying both OMCN and thyroid cancer cases, while standard practice, introduces potential classification errors. The analysis was further constrained by our inability to assess disease severity, specific subtypes, or treatment protocols, factors that could significantly influence outcomes and potentially affect the interpretation of our findings. Additionally, while we adjusted for numerous variables, the observational nature of our study leaves room for unmeasured confounding factors. These include family medical history, environmental exposures, dietary patterns, and ongoing medication use. The dynamic nature of health behaviors and medical interventions over time presents another analytical challenge, as our study could not fully account for these temporal changes. To address potential reverse causation—where undiagnosed thyroid cancer might precipitate OMCN—we implemented a one-year lag period in our analysis. However, this approach may not completely eliminate this source of bias. Finally, external validity represents another limitation of our study. Given that we analyzed data exclusively from a Korean population, caution is warranted when extending these findings to other ethnicities or healthcare systems. Confirming these relationships in diverse populations through international collaborative studies would enhance the global relevance of our observations.

In conclusion, this nationwide large-scale study provides novel evidence of an association between OMCN and thyroid cancer risk. Thus, referrals for thyroid cancer evaluation should be considered, especially in diabetic women diagnosed with OMCN. In addition, given the commonly compromised cardiovascular system present in OMCN and thyroid cancer and the observed association between OMCN and thyroid cancer, the diagnosis of OMCN should prompt discussions on lifestyle modifications and interventions aimed at addressing these cardiovascular risk factors to possibly reduce the thyroid cancer risk. This research opens a new avenue for a deeper understanding of the systemic implications of OMCN and establishes a basis for subsequent studies focused on early detection and prevention of thyroid cancer in OMCN patients. Additional research is needed to validate the association between OMCN and thyroid cancer and to thoroughly investigate the underlying pathophysiological mechanisms related to cardiovascular risk factors.
